# Allergens in Food: Analytical LC-MS/MS Method for the Qualitative Detection of *Pistacia vera*

**DOI:** 10.3390/foods14173031

**Published:** 2025-08-29

**Authors:** Roberta Giugliano, Sara Morello, Samantha Lupi, Barbara Vivaldi, Daniela Manila Bianchi, Elisabetta Razzuoli

**Affiliations:** 1 Istituto Zooprofilattico Sperimentale del Piemonte, Liguria e Valle D’Aosta, 16129 Genoa, Italy; roberta.giugliano@izsplv.it (R.G.); barbara.vivaldi@izsplv.it (B.V.); elisabetta.razzuoli@izsplv.it (E.R.); 2 Istituto Zooprofilattico Sperimentale del Piemonte, Liguria e Valle D’Aosta-SC Sicurezza Alimentare, 10154 Turin, Italy; sara.morello@izsplv.it (S.M.); samantha.lupi@izsplv.it (S.L.); manila.bianchi@izsplv.it (D.M.B.); 3 Istituto Zooprofilattico Sperimentale del Piemonte, Liguria e Valle D’Aosta-Centro di Referenza Nazionale per la Rilevazione Negli Alimenti di Sostanze e Prodotti che Provocano Allergie e Intolleranze-CReNaRiA, 10154 Turin, Italy

**Keywords:** food allergens, pistachio, LC-MS/MS analysis

## Abstract

Pistachio (*Pistacia vera*) is widely consumed among tree nuts but capable of triggering severe IgE-mediated reactions in allergic individuals. Due to the similarity of cashew-borne and pistachio-borne allergen proteins and DNA, traditional detection methods, such as ELISA and PCR, often suffer from cross-reactivity, limiting their ability to discriminate between these two allergens. This study presents a sensitive LC-MS/MS method for the simultaneous detection of pistachio and cashew allergens in processed food with a screening detection limit (SDL) equal to 1 mg/kg. The method was validated for specificity, SDL, β error, precision, and ruggedness, and applied to various matrices (cereals, chocolate, sauces, and meat products). Ruggedness testing showed that all considered parameters must be carefully monitored by the operator, and sample preparation must be carried out without any modification in parameter values, under strictly controlled conditions. Good reproducibility was achieved for pistachio detection, while ongoing investigations should be carried out to overcome existing constraints for cashew. The LC-MS/MS method described in this work is a discriminatory method suitable for official food allergen control to selectively differentiate pistachio from cashew allergens, overcoming the limitations of PCR and ELISA when cross-reactivity occurs. It represents a validated tool for pistachio detection and a promising approach toward improving cashew allergen analysis.

## 1. Introduction

Among tree nuts, the pistachio (*Pistacia vera*) belongs to the *Anacadiaceae* family, as does the cashew (*Anacardium occidentale* L.) [1,2]. According to the information available on the United States Department of Agriculture’s Germoplasm Resources Information Network, the genus *Pistacia* encompasses at least 12 different species, some of which have different subspecies [3]. The genus *Anacardium* comprises 11 species, with *Anacardium occidentale* L. (cashew) being the most common in Brazil, particularly in the northern and northeastern regions. The most important producing countries are Iran, California, the Middle East, India, Greece, and Pakistan [4]. Pistachios and cashews are widely considered to be important foods with high nutritional value due to their potential health benefits, as they are a source of healthy lipids, fibre, protein, antioxidants, vitamin B6, vitamin B1, and potassium [5]. However, they can also pose a threat to consumers’ health due to their potential harmful effects, as they are widely recognized as allergen-containing foods. Trace amounts of undeclared pistachio and cashew allergens can pose serious health risks to consumers with food allergies, who should avoid consuming these foods or foods containing traces of them [6,7]. In the food industry, the risk of unintentional cross-contamination may occur during food production and storage [8]. Some of the proteins included in their proteome can trigger severe IgE-mediated reactions and anaphylaxis in allergic individuals. Typical symptoms of an allergic reaction to tree nuts include itching, swelling, rashes, breathing difficulties, diarrhea, vomiting, and anaphylactic shock [9]. Around 1.1% of the European adult population currently has an allergy to these two tree nuts, and 7% of reported cases of tree nut allergy were caused by pistachios [10,11,12,13].

The General Food Law (Regulation (EC) No 178/2002) of the European Union requires that food business operators establish proper risk assessment and management procedures to guarantee the safety of food on the market [14]. According to Regulation (EU) No 1169/2011, tree nuts represent 1 of 14 substances that can cause food intolerance or allergy [15,16]. Regulation (EU) No 1169/2011 does not establish a defined threshold or limits for food allergens, except for gluten, whose limit is set in a separate regulation [15,16]. Consequently, pistachios and cashews must be declared as allergens on food labels when used as ingredients. In some situations, food business operators may be unable to prevent or reduce the unintended presence of allergens. In such cases, they can use voluntary Precautionary Allergen Labelling (PAL) as a means of communication and risk management. Regulation (EU) No 1169/2011 provides the legal basis for using PAL, stating that “voluntary information shall not be ambiguous or confusing for the consumer, and shall be based on the relevant scientific data” [15,16]. However, the absence of specific guidance on the application of PAL leads to its varied and inconsistent use. Consequently, consumers with food allergies lose trust in PAL, reducing its effectiveness in protecting against food allergens [16].

The two major techniques employed for food allergen detection are polymerase chain reaction (PCR) and immunoassays such as the enzyme-linked immunosorbent assay (ELISA) [1,5,8,17,18,19,20,21]. Currently, five allergenic proteins are officially listed for the pistachio nut (*Pis v1*, *Pis v2*, *Pis v3*, *Pis v4*, and *Pis v5*), while three groups of allergenic proteins have been identified and characterized in the cashew nut (*Ana o1*, a vicilin protein; *Ana o2*, a legumin protein; and *Ana o3*, a 2S albumin) [22,23,24,25]. The pistachio allergens identified so far belong to the protein families of 2S albumin (*Pis v1*), legumin (*Pis v2* and *Pis v5*), vicilin (*Pis v3*), and iron/manganese superoxide dismutase (*Pis v4*).

Due to the similarity of the two cashew- and pistachio-borne allergen proteins and DNA, some ELISA and PCR methods may not be able to distinguish between the two sources of contamination. Multianalyte sensitive and robust methods have been developed and validated in-house to help laboratories discriminate between pistachio and cashew contamination [26,27,28,29].

The recent literature on nut allergies remains relatively scarce, regarding updated clinical data and population-specific patterns. This absence of recent comprehensive studies highlights a gap in current knowledge and underscores the need for further research in this area [26,27,28,29]. Different analytical methods have been developed for food allergen detection, as outlined by Van Hengel et al. [30]. To date, the most employed techniques in official food allergen analysis remain the enzyme-linked immunosorbent assay (ELISA) and the polymerase chain reaction (PCR). However, both techniques have significant limitations. ELISA which relies on antigen–antibody interactions, is susceptible to cross-reactivity with food matrix components, potentially resulting in false positives [31,32]. Furthermore, ELISA is generally limited to single-target detection, and robust multiplexing has yet to be fully established [33,34]. In contrast, PCR targets allergen-specific DNA sequences rather than the allergenic proteins (or peptides) themselves. This indirect approach may lead to inaccurate results, particularly in processed foods, where proteins and DNA may degrade and become physically separated during manufacturing [35,36].

Mass spectrometry (MS)-based techniques have recently become a powerful tool for the analysis of food allergens due to their high sensitivity and ability to provide unequivocal allergen identification [29,37,38,39,40,41]. MS has some advantages over the techniques described above. Unlike ELISA, it is unaffected by antibody cross-reactivity, and unlike PCR, it allows for the direct detection of allergenic peptides or proteins, ensuring more accurate identification. Furthermore, MS enables a multi-target approach, simultaneously detecting different analytes in a single chromatographic run. In particular, in LC-MS/MS, isotopically labelled internal standards and label-free approaches can be used for quantification [37,38,41]. Isotopically labelled standard proteins/peptides are widely used in most published studies due to their ability to be added prior to the extraction and/or digestion. However, these labelled standards can be costly, making label-free proteins/peptides a more practical alternative [37,38,41]. Recently, published approaches have adopted external calibration curves or standard addition methods [42]. Multianalyte sensitive and robust methods have been developed and validated in-house to support laboratories in discriminating between pistachio and cashew. Nevertheless, there is still no harmonization of methodological regulation through globally accepted guidelines. Despite these advantages, MS remains a high-cost and time-consuming technique and requires skilled personnel for method development and routine operation.

Among the various analytical methods reported in the literature [13,26,27,37,42,43,44,45], the protocol developed by Sealey-Voyksner et al. in 2016 [42] uses LC-MS/MS with a q-TOF detector to detect allergens. The detector q-TOF enables the scanning and detection of a wider range of masses than triple quadrupole mass spectrometry (LC-QqQ). The method proposed by Sealey-Voyksner et al. in 2016 [42] leverages the presence of both allergenic proteins and protein fragments obtained through enzymatic digestion. The main advantage of using QqQ in official food control laboratories lies in its suitable platform for official food control laboratories due to its sensitivity, specificity, and reproducibility in targeted analysis. Moreover, LC-QqQ instrumentation is widely implemented in food control laboratories, offering a cost-effective and robust solution that is compatible with routine high-throughput workflows and method standardization. However, while most food control laboratories use LC-QqQ, few have q-TOF detectors. Currently, all food control laboratories require an analytical method capable of verifying the presence of pistachio or cashew nuts, particularly in case where the ELISA and PCR methods show cross-reactivity and cannot distinguish between the presence of the two allergens.

Our study aims to contribute to closing this gap by providing new data and insights regarding the detection of allergens in food using updated methods, in line with the mission of the National Reference Centre for the detection of substances and products causing allergies or intolerances in food. Indeed, in the present study, we aimed to develop an analytical method for the simultaneous detection of pistachio and cashew allergens in foods using liquid chromatography–mass spectrometry, with the goal of establishing a discriminatory chemical method able to selectively identify these two allergenic nuts using an LC-QqQ.

The study design is detailed in the Materials and Methods, where we describe the sample collection (Section 2.1 ) and the sample preparation (Section 2.3). Then, we present the technical parameters adopted for the LC-MS/MS analysis in Section 2.4. PCR and ELISA analysis are described in Section 2.6 . The development of the LC-MS/MS technical parameters analysis and the validation parameters investigated in this study (specificity, screening detection limit (SDL), β error, precision, and ruggedness) are reported in Section 2.5 .

## 2. Materials and Methods

### 2.1. Sample Collection

For the validation tests, an initial assessment of potential allergen contamination was carried out on 20 representative samples (4 cereal-based products, 4 chocolate-based products, 3 sauces, 3 meat-based products, 3 beverages, and 3 milk-based products). All samples were purchased from several retail stores in the Liguria and Piedmont regions (Italy). The selected samples did not contain pistachios and cashews in either the PAL or the ingredients list. Precautions were taken to avoid the purchase of similar products with the same brand name from different stores.

Therefore, the laboratory collected and stored 3 naturally contaminated samples with allergens, i.e., *Pistacia vera* and *Anacardium occidentale* L. (1 bakery product, 1 cocoa, and 1 olive pate), and 2 samples involved in laboratory Proficiency Testing (PT), containing a certified quantity of *Pistacia vera* and *Anacardium occidentale* L. (Fera Science Ltd., Sand Hutton, York, UK).

All samples described in this subsection were previously analyzed using validated and accredited PCR and ELISA methods for the detection of pistachio and cashew, respectively (see Section 2.6). No positive results were observed in the 20 samples used as blank for the validation study; meanwhile, the 3 naturally contaminated and PT samples tested positive using PCR and ELISA. All the samples were stored at a temperature of 5 ± 3 °C until analysis.

### 2.2. Reagents and Chemicals

Synthetized peptides (QLQQQEQIK for the protein *c1* of *Anacardium occidentale* L. and LQELYETASELPR for the protein *Pis v1* of *Pistacia vera*) were purchased from EspiKem (Prato, Italy). Stock solutions of each peptide were prepared in water at 100 mg/L and stored in the freezer at (−30 ÷ −15) °C. Acetonitrile, methanol, and water were purchased from Carlo Erba (Milan, Italy). Trifluoroacetic acid (TFA, degree of purity ≥ 99%) was purchased from Panreac AppliChem (Milan, Italy). Trypsin, Bovine Pancreas (degree of purity ≥ 90%), TRIS HCl (degree of purity ≥ 99%), and Na_2_HPO_4_ (degree of purity > 96%) were purchased from Merck (Sigma-Aldrich KGaA, Darmstandt, Germany). All reagents were of analytical grade. Additionally, an Eppendorf™ 5427 R microcentrifuge (Hamburg, Germany) and a 37 ± 2 °C Memmert™ thermostatic incubator (Memmert GmbH & Company KG, Buchenbach, Germany) were used.

### 2.3. Sample Preparation

The procedure for sample and working solution preparation and protein extraction published by Sealey-Voyksner and collaborators was applied with slight modifications [42]. Firstly, solutions of 50 mM TRIS/HCl (pH 7.5), 50 mM dibasic sodium phosphate (pH 8), 12 mg/mL trypsin, and 0.2% TFA in acetonitrile were prepared. The TRIS/HCl solution was prepared by weighing 79 ± 1 mg of TRIS/HCl on an analytical balance and dissolving it in 10 mL of ultrapure water. The dibasic sodium phosphate solution was prepared in the same manner, by weighing 71 ± 1 mg of the compound and dissolving it in 10 mL of ultrapure water. The trypsin solution was prepared by dissolving 120 ± 1 mg of trypsin in 10 mL of the dibasic sodium phosphate solution. Finally, the 0.2% TFA acetonitrile solution was prepared by diluting 20 µL of TFA solution in 10 mL of acetonitrile.

The samples were ground to a fine powder. Then, the proteins from 30 mg ± 0.001 g of each sample were extracted for 2 h at 50 °C with 1000 µL of the TRIS/HCl solution. Enzymatic digestion was conducted on this extract by adding 25 µL of the trypsin solution at 38 °C for 2 h, vortexing each sample every 15 min. After this time, the reaction was stopped by adding 200 µL of 0.2% TFA in acetonitrile. Then, the sample was centrifuged at 7871 g for 10 min and the supernatant was dispensed in a vial and injected into the LC-MS/MS system.

### 2.4. Instrumental Conditions for LC-MS/MS Analysis

For the detection, an HPLC Accela coupled with a TSQ Vantage (Thermo Fisher Scientific, Waltham, MA, USA) was employed. Mobile phases consisted of (A) 0.025% TFA in aqueous solution ACN:H_2_O (95:5 *v*/*v*) and (B) 0.025% TFA in ACN:H_2_O (5:95 *v*/*v*). The flow rate was set at 250 μL/min and the injection volume was 10 µL.

The gradient elution was as follows: from 0.0 min to 40.0 min at 75% of A, and from 40.0 min to 45.0 min at 40% of A, at the last re-equilibration at initial conditions for 5 min.

Chromatographic separation was achieved on SecurityGuard ULTRA cartridges for EVO-C18; UHPLC (sub-2 μm and core–shell columns with ID = 2.1 mm) and a column LC Kinetex^®^ (2.6 μm C18 100 Å 50 × 2.1 mm, Phenomenex, Torrance, CA, USA) were used.

The voltage of the Heated Electrospray Ionization (HESI) source was set at −2500 ÷ 3500 V, the capillary temperature at 300 °C, the vaporizer temperature at 200 °C, and the collision gas pressure at 1.2 mTorr.

The analysis was conducted in Single Reaction Monitoring (SRM) to enhance selectivity and sensitivity for the analytes by selecting two precursor ions and their corresponding product ions based on their excellent intensity.

Detailed values for the precursor ions and product ions, along with the collision energies for the analytes, are listed in Table 1.

### 2.5. LC-MS/MS Method Optimization and Validation

Utilizing a Heated Electrospray Ionization (HESI) interface, the instrumental parameters were optimized using the stock solutions of each peptide, infused directly into the mass spectrometer, bypassing chromatographic separation. The best tuning conditions were identified for acquiring the SRM signals of the most stable and significant transitions of the analytes of interest and for the maximum sensitivity of the protonated molecular ion. Three SRM transitions were optimized and selected for each compound, as reported in Table 1.

Validation procedures were performed following the EURL guidance document on the quality control during routine analysis (ongoing method performance verification) [46] and the EURL Guidance Document on Screening Method Validation [47]. The protocol of LC-MS/MS method validation includes the following performance characteristics: specificity, SDL, β error, precision, and ruggedness.

The specificity of the method was assessed by injecting blanks (n = 20) and fortified samples (n = 20) in two independent analytical sessions by LC-MS/MS. Blank samples were fortified using stock solution of each peptide to obtain the final concentration in a matrix of 1 mg/kg. The absence of a signal-to-noise (S/N) ratio below 3 at the retention time ranges (tR sample = tR working solution ± 0.25 min) of the target compound indicated that the method was free of interference. Conversely, the presence of signal was evaluated if the S/N ratio was above 10 within the retention time range.

For a qualitative screening method, it is required that the detection of the analyte can be reliably established at a defined concentration level, referred to the screening detection limit (SDL). The SDL is the lowest concentration level at which the analytes could be identified in all fortified samples, in at least 95% of the cases. The SDL was determined by testing 20 samples fortified at the fortification level of 1 mg/kg. Since any limit for *Pistacia vera* and *Anacardium occidentale* L. is established in the Regulation (EU) No 1169/2011 [15,16], during the validation test the fortification level adopted was chosen to be equal to the MS-based SDL described above.

The estimation of the β error was conducted following the Regulation (EU) No 808/2021, “the β error is the probability of a false negative result in screening methods. The regulation mandates that screening methods must reliably detect the substance of interest at or below the level of interest, with a false compliant rate (β error) not exceeding 5%” [48]. In this study, the β error was calculated on 20 samples fortified (at 1 mg/kg) and we accepted a β error less than or equal to 5% (i.e., 1 sample for each peptide).

The precision of the method was evaluated by calculating the coefficient of variation (CV%) on the results obtained from 20 samples fortified at the fortification level of 1 mg/kg of each peptide.

Ruggedness was evaluated using the Youden approach, considering the following parameters: centrifuge force and time, the digestion temperature and time, the volume of trypsin, TRIS HCl, and acetonitrile [49]. The ruggedness assesses, within a single laboratory, how sensitive analytical methods are to slight variations in method parameters, using experimental designs. These preliminary tests are more cost-effective than collaborative studies and enable adjustments to the method before it undergoes collaborative evaluation. Experimental conditions are summarized in Table 2.

To test the applicability of the method in routine, LC-MS/MS analyses were performed on the 3 naturally contaminated and the 2 PT samples stored (Section 2.1).

Routine quality control involves monitoring the peak areas of fortified sample injected during every batch using quality control charts. After entering 20 data in the preliminary step, the spreadsheet automatically calculates the mean value and control ranges (average ± 2 SD and average ± 3 SD; SD = standard deviation). The monitored values track the performance of the method over time and control that the detected values fall within the defined uncertainty range.

### 2.6. PCR and ELISA Analysis

PCR and ELISA analyses were performed on the samples listed in Section 2.1.

To determine the presence of the pistachio allergen, PCR analysis was performed following an accredited in-house method. The method included DNA extraction from 200 mg of sample using 100 µL of proteinase K in 900 µL of QIAamp DNA Mini Kit buffer. The extraction was followed by overnight incubation at 56 °C, vortexing at 241 g, and transferal into a 2 mL Eppendorf containing cold isopropanol (−20 °C). The solution was then centrifugated again, and the precipitate resuspended in 100 µL of QIAamp buffer.

One reverse and one forward primer for the pistachio were as follows: Pist F2 (5′-GAA ATC TTA ACG AGA GAG CTC GCT-3′) and Pist R2 (5′-CGT TGC CGA GAG TCG TTA TTG-3′); the probe used was PistTM1Rev (FAM-CTA CCC ATC CCG CAC GCG C-BBQ). PCR was carried out in 10 µM of primer and probe solution, and after an initial denaturation step at 95 °C for 480 s, the PCR conditions were optimized as follows: 40 cycles at 95 °C; 60 °C, 10 s; 72 °C, then at 50 °C for 30 s. Threshold cycle of quantification ≤ 28. Quality control samples were prepared following the same protocol as the samples starting from 1 g of *Pistacia vera*.

To determine the presence of the cashew allergen, ELISA analyses were performed following the IMMUNOLAB Cashew ELISA kit instruction (Oxford BioSystem^®^, Abingdon, UK). Briefly, the extraction phase was performed from 1 g of sample. The 10 mg/kg positive quality control sample was prepared by fortifying a blank food sample using a 100 mg/L spiking solution. The spiking solution was obtained from a 50.000 mg/L stock solution previously prepared and subjected to serial dilutions.

The extraction solution (20 mL) provided by the kit was used, followed by incubation at 60 °C for 15 min and subsequent centrifugation. For the ELISA, standards, sample extracts, and controls were dispensed into the microplate and incubated at room temperature for approximately 30 min. The incubation phase with the enzyme was followed by incubation with a chromogen/substrate solution and subsequent optical density reading at 450 nm.

## 3. Results

### 3.1. LC-MS/MS Validation

A reliable analytical LC-MS/MS qualitative method was fully validated in-house considering the following performance characteristics: specificity, SDL, β error, precision, and ruggedness.

Specificity was confirmed by the absence of interfering peaks at the retention time ranges (tR sample = tR working solution ± 0.25 min) of each target compound. In all blank samples analyzed, the S_b_/N ratio was below 3. The presence of signal was confirmed with an S_f_/N ratio above 10 in all fortified samples at the concentration of 1 mg/kg. Areas of the fortified samples are listed in Table S1 of the Supplementary Information.

For both *Pistacia vera* and *Anacardium occidentale* L., the SDL was determined as being equal to 1 mg/kg and was identified in at least 95% of all fortified samples (n = 20).

As reported in Table S1 of the Supplementary Information, for *Pistacia vera* we detected the peptide LQELYETASELPR in 19 out of 20 fortified samples; meanwhile, for *Anacardium occidentale* L. we detected the peptide QLQQQEQIK in all 20 fortified samples. Thus, for *Pistacia vera* we calculated a β error equal to 5% and for *Anacardium occidentale* L. a β error less than 5%.

The precision of the method showed a CV% equal to 26% for *Pistacia vera* and 50% for *Anacardium occidentale* L. at the SDL of 1 mg/kg.

The results obtained during the ruggedness testing revealed that all parameters reported in Table 2 must be carefully monitored by the operator. Indeed, by strictly following the sample preparation described in Section 2.3, without any deviation from the values detailed in Table 2, the analysis showed good reproducibility in all fortified samples, even in the case of roasted and unroasted matrices.

### 3.2. Applicability of the Method for Routine Analysis

**Figure 1:**
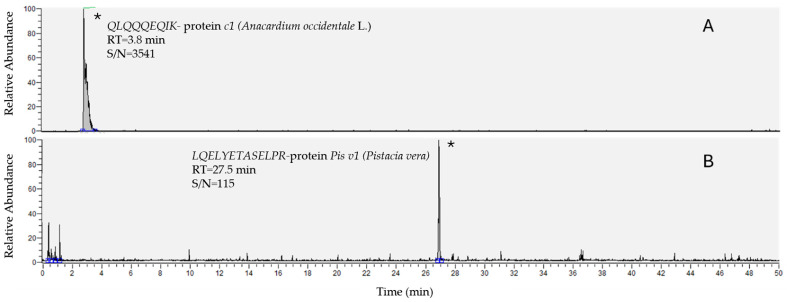
TICs (total ion chromatograms) of QLQQQEQIK for the protein *c1* of *Anacardium occidentale* L. (**A**) and LQELYETASELPR for the protein *Pis v1* of *Pistacia vera* (**B**). The signals (*) refer to the fortified samples of cereal-based products at 1 mg/kg.

**Figure 2:**
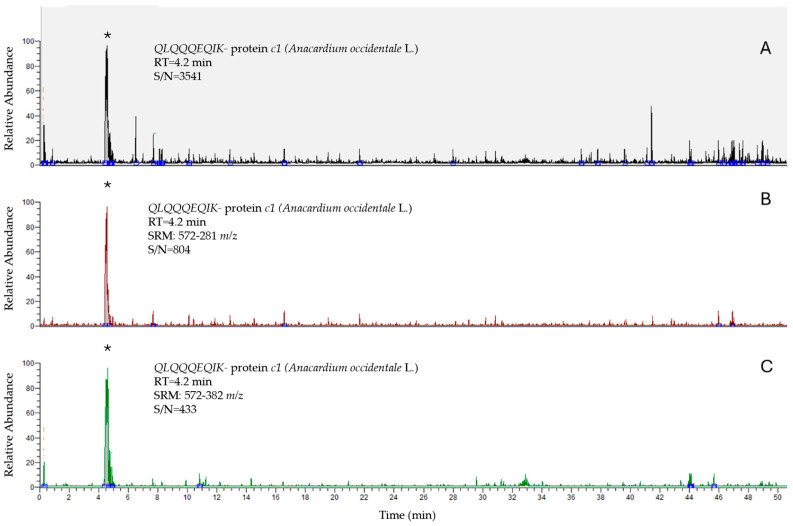
Chromatograms of QLQQQEQIK for the protein *c1* of *Anacardium occidentale* L. in cereal-based products: TIC (**A**); SRM: 572-281 *m/z* (**B**); SRM: 572-382 *m/z* (**C**). The signals (*) refer to the fortified sample at 1 mg/kg.

**Figure 3:**
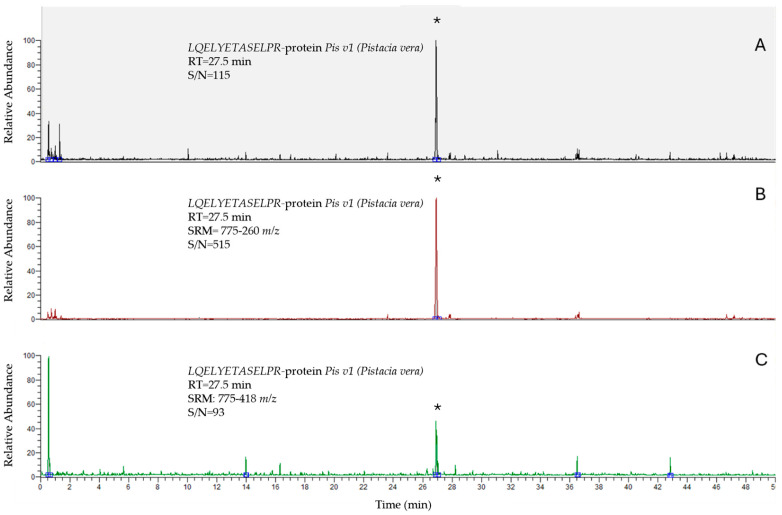
Chromatograms of LQELYETASELPR for the protein *Pis v1* of *Pistacia vera* in cereal-based products: TIC (**A**); SRM: 775-260 *m/z* (**B**); SRM: 775-418 *m/z* (**C**). The signals (*) refer to the fortified sample at 1 mg/kg.

The analysis of the three naturally contaminated and the two PT samples was performed using the validated method described in Section 2 Materials and Methods. Chromatograms are reported in Figure 1, Figure 2 and Figure 3.

Meanwhile, the analysis previously performed on naturally contaminated samples using ELISA and PCR methods revealed cross-reactivity; LC-MS/MS was successively carried out and allowed the distinction between the two allergens. All results obtained in routine analysis are reported in Table 3.

## 4. Discussion

As members of the same *Anacadiaceae* family, pistachio and cashew plants exhibit similar protein expression profiles in their kernels. This molecular similarity often results in cross-allergy, with many allergic individuals exhibiting simultaneous sensitivity to both nuts. Numerous studies have confirmed the significant cross-reactivity observed in commonly used techniques (ELISA and PCR) when detecting the IgE-binding proteins [13,26,27,37,42,45,50,51,52,53] Although studies focusing specifically on distinguishing between these two closely related allergens remain limited, recent research has demonstrated the effectiveness of mass spectrometry (MS)-based techniques in overcoming the limitations of ELISA and PCR in this respect. Our results align with these observations, and in the present study we propose an MS-based method to try to overcome this limitation through careful peptide selection and method optimization [42]. Several studies have explored MS-based analytical approaches, particularly in the analysis of complex food matrices and we compared our data with that from similar studies conducted in recent years [13,26,27,37,42,43,44,45].

The study by New et al. focused on developing a method to simultaneously detect multiple allergens, including pistachio and cashew, in food products using triple quadrupole Q-Trap mass spectrometry [43]. While their work demonstrated the feasibility of multi-allergen detection using an MS approach, it did not specifically address the analytical challenge of discriminating between these two allergens when ELISA and PCR methods showed cross-reactivity [43]. Korte et al. introduced an advanced multiple reaction monitoring cubed (MRM^3^) strategy aimed at improving both sensitivity and specificity in nut allergen detection [54]. However, while this method offers excellent analytical performance and enhanced selectivity, it may not be easily applicable in routine food testing laboratories due to the complexity of the acquisition mode and the need for more sophisticated instrumentation [54].

High-resolution MS approaches have also made a significant contribution to the field. Recently, Luparelli et al. developed a high-resolution method for multi-analyte determination of tree nuts and peanuts in processed food matrices [44]. This method has proven effective in characterizing multi-allergen profiles and represents an important advance in allergen detection [44]. However, the complexity and operational costs of high-resolution instruments limit their routine use in official food control settings [44].

Sealey-Voyksner et al. used q-TOF-based profiling to analyze tree nut allergens and discovered a highly conserved tree nut peptide that could selectively detect the type of nut it came from [42]. In their study, a multi-allergen detection method was performed using a q-TOF mass spectrometer. However, even with such a well-designed method, adopting a q-TOF-based analytical approach can be challenging for routine laboratories, as most are equipped with QqQ mass spectrometers.

Compared to methods reported in the literature, the analytical approach presented here is faster and more cost-effective, as it does not require the use of isotopically labelled standards [13,26,27,37,45]. Our work presents a targeted approach specifically optimized and validated for the discrimination between pistachio and cashew allergens using QqQ mass spectrometry. Our results are comparable with the findings of Sealey-Voyksner et al., who analyzed unroasted peanuts, peanut butter, protein bars, and nut crisps, and found all the investigated markers in all these matrices [42]. For our purposes, the key difference between these two instruments lies in their *m/z* range. Specifically, q-TOF can detect ions with higher *m/z* values than the QqQ instrument. Consequently, q-TOF detectors can detect the protein and/or the heaviest peptide, whereas QqQ instruments can only detect the lightest ones [55].

To assess the validation, the authors verified specificity, SDL, β error, precision, and ruggedness following the *EURL guidance document on the quality control during routine analysis (ongoing method performance verification)* [46] and *the EURL Guidance Document on Screening Method Validation* [47].

For the validation of the pistachio MS-based method [42], the authors adopted the chromatographic peak of the fortified samples as the signal for parameter verification. Meanwhile, for cashew nut validation, the signal was defined as the analyte peak in the fortified sample minus the peak in the blank sample. This adjustment was necessary due to the high polarity of the target cashew peptide, which elutes early in the chromatogram, where matrix interferences are much more significant compared to the rest of the chromatogram. However, our specificity results highlighted the good capability of the method to accurately distinguish the two nut signals, even at very low SDLs with a β error below 5%.

Matrix interferences pose challenges for peak identification and integration, affecting the precision of the method, as reflected by the high cashew CV% (Table S2 in the Supplementary Information). Several ongoing investigations aim to better understand and improve this limited reproducibility in cashew analysis. Preliminary data suggest that matrix interferences could be minimized by introducing a fortified matrix similar to the sample matrix, but further studies are necessary to explore other potential factors affecting cashew analysis reproducibility, such as environmental conditions or instrumental variables. The development of quantitative Certified Reference Materials (CRMs) for MS-based analysis could facilitate these investigations. On the other hand, pistachio precision does not appear to be significantly affected by interferences (Table S2 in the Supplementary Information).

The Youden test highlighted the poor ruggedness of the method (Table 2). Our data revealed that while pistachio detection maintains good reproducibility when all analytical parameters are kept within specified ranges, the same does not apply to cashew analysis (Table 2). Indeed, our MS-based method requires qualified and highly experienced technicians to ensure reliable application.

Overall, this study presents a fully validated pistachio detection protocol suitable for official food control, and provides preliminary data for cashew detection and validation, which require further investigation to better understand the limitation of the protocol.

## 5. Conclusions

The proposed LC-MS/MS protocol enables critical discrimination between pistachio and cashew as food allergens—overcoming ELISA and PCR cross-reactivity—providing a sensitive and more accessible technique suitable for allergen testing in food control laboratories, compared to other MS-based protocols [13,26,27,37,45].

The novelty of our study lies in the validation of a targeted LC-MS/MS method for pistachio detection using a QqQ mass spectrometer—an analytical instrument widely available in official food control laboratories. In contrast to previously published methods employing Q-TOF instruments, our QqQ-based method is more suitable for routine targeted detection due to its superior sensitivity, reproducibility, and cost-effectiveness.

In conclusion, the present study describes a fully validated protocol for *Pistacia vera* identification, highlights limitations encountered during cashew identification. Our MS-based method offers a validated solution for pistachio detection and a promising foundation for use in scenarios where traditional methods such as PCR and ELISA exhibit cross-reactivity between pistachio and cashew, limiting their specificity.

## Figures and Tables

**Table 1:** SRM scan parameters: precursor and product ion (*m/z*), voltage of collision energy (V) and S-lens (V).

Peptide	Polarity	Precursor Ion (*m/z*)	Product Ion (*m/z*)	Collision Energy (V)	S-Lens (V)
QLQQQEQIK	+	572	281	53	125
		572	382	34	125
		572	563	20	136
LQELYETASELPR	+	775	260	50	120
		775	418	47	120
		775	542	17	120

**Table 2:** Parameters monitored for the evaluation of the ruggedness using the Youden approach.

Parameters	Values	Value Ranges
Centrifugation force	7871 g	6375–9524 g
Centrifuge time	10 min	9–11 min
Digestion temperature	38 °C	34.2–41.8 °C
Digestion time	120 min	108–132 min
Trypsin volume	25 µL	22.5–27.5 µL
TRIS HCl volume	1000 µL	900–1100 µL
Acetonitrile volume	200 µL	180–220 µL

**Table 3:** LC-MS/MS results obtained in routine samples.

	*Pistacia vera*		*Anacardium occidentale* L.	
Matrix	tR (min)	Sample (Area)	tR (min)	Sample (Area)
Bakery product	26.8	967	/	not detected
Cocoa	/	not detected	3.8	1452
Olive Sauce	/	not detected	/	not detected
PT cashew	/	not detected	3.9	1013
PT pistachio	27.1	927	/	not detected

## Data Availability

The original contributions presented in the study are included in the article, and further inquiries can be directed to the corresponding author.

## Supplementary Materials

The following supporting information can be downloaded at: https://www.mdpi.com/article/10.3390/foods14173031/s1, Table S1: Areas of the validation session for *Pistacia vera* and *Anacardium occidentale* L. matrices; no detectable signals were identified in any of the blank matrices; Table S2: Results of the validation performance characteristics.

## Abbreviations

The following abbreviations are used in this manuscript:

LC-MS/MS	Liquid Chromatography–Tandem Mass Spectrometry
CRMs	Certified Reference Materials
SRM	Single Reaction Monitoring
TFA	Trifluoroacetic acid
ACN	Acetonitrile
ELISA	Enzyme-Linked Immunosorbent Assay
PCR	Polymerase Chain Reaction
TOF	Time-Of-Flight
QqQ	Triple Quadrupole
tR	Retention Time
PT	Proficiency Testing
CV%	Coefficient of Variation
SD	Standard Deviation
SDL	Screening Detection Limit
S/N	Signal-to-Noise
HESI	Heated Electrospray Ionization
HPLC	High-Performance Liquid Chromatography
PAL	Precautionary Allergen Labelling
MRM^3^	Multiple Reaction Monitoring Cubed
IgE	Immunoglobulin E
