# Supplementary material for: Allergens in Food: Analytical LC-MS/MS Method for the Qualitative Detection of Pistacia vera
Source: Foods. 2025 Aug 29;14(17):3031. doi: 10.3390/foods14173031 (PMC12428185; doi:10.3390/foods14173031)
Supplement: Supplementary file 1 [file foods-14-03031-s001.zip › foods-3794395-supplementary.pdf]

Supplementary Information

Table S1: Areas of the validation session for *Pistacia vera* and *Anacardium occidentale* L. matrices, in all blank matrices no detectable signals were identified.

	Pistacia vera		***Anacardium occidentale* L.**	
**Matrix**	tR(min)	Fortified sample (area)	tR (min)	Fortified sample (area)
**Cereal-based products**	26.9	1036	3.8	50048
**Chocolate-based products**	26.8	1023	3.8	53749
**Sauces**	26.8	961	3.8	55410
**Meat-based products**	26.7	957	3.8	59877
**Beverages**	26.8	888	3.8	61821
**Milk-based products**	26.7	886	3.9	57929
**Cereal-based products**	26.5	1050	4.2	61831
**Chocolate-based products**	26.5	903	3.8	59494
**Sauces**	26.6	1291	3.8	1107
**Meat-based products**	26.7	not detected	3.7	701
**Beverages**	26.8	974	3.8	52657
**Milk-based products**	26.7	994	3.8	59503
**Cereal-based products**	26.7	1083	3.9	54569
**Chocolate-based products**	26.8	1098	3.8	59625
**Sauces**	26.7	890	3.9	57813
**Meat-based products**	26.6	1032	3.8	54525
**Beverages**	26.6	1093	3.8	56295
**Milk-based products**	26.6	852	3.8	51511
**Cereal-based products**	26.5	1203	3.8	948
**Chocolate-based products**	27.5	1102	4.2	632

Table S2. Results of the validation performance characteristics.

	**Results**	
**Validation performance characteristics**	*Pistacia vera*	*Anacardium occidentale* L.
**Specificity (S_b/N<3 & S_f/N>10)**	Verified	Verified
**SDL (mg/kg)**	1	1
**β error (%)**	5	≤5
**Precision (CV (%))**	26	50
**Ruggedness**	Poor ruggedness	Poor ruggedness
